# ASSOCIATION BETWEEN SERUM BIOMARKERS OF ALZHEIMER’S DISEASE AND “DUAL DECLINE” IN COGNITIVE AND MOTOR FUNCTIONS

**DOI:** 10.1093/geroni/igae098.2014

**Published:** 2024-12-31

**Authors:** Elena Pinardi, Alice Ornago, Giulia Grande, Martina Valletta, Debora Rizzuto, Erika Jonsson Laukka, Giuseppe Bellelli, Davide Liborio Vetrano

**Affiliations:** Karolinska Institutet, Italy; Karolinska Institutet, Osnago, Lombardia, Italy; Karolinska Institutet, Stockholm, Stockholms Lan, Sweden; Karolinska Institutet, Stockholm, Stockholms Lan, Sweden; Karolinska Institutet, Stockholm, Stockholms Lan, Sweden; Karolinska Institutet, Stockholm, Stockholms Lan, Sweden; University of Milano-Bicocca, Milano, Lombardia, Italy; Karolinska Institutet, Stockholm, Stockholms Lan, Sweden

## Abstract

Individuals experiencing simultaneous decline in cognitive and motor performances (i.e., dual decliners) face an increased risk of dementia and other adverse health outcomes. While the underlying pathophysiology remains largely unknown, this study aims to explore the association between serum biomarkers of Alzheimer’s disease (AD) and various patterns of cognitive/motor decline. Data are drawn from the Swedish National Study on Aging and Care in Kungsholmen. The rates of cognitive and motor decline were assessed over a 15-year period using linear mixed models, with Mini-Mental State Examination and gait speed as the respective measures. Participants were categorized into slow/non-decliners (reference group, n=1115), fast motor decliners (n=162), fast cognitive decliners (n=162), and dual decliners (n=264). The association between serum AD markers levels and the four decline patterns was analyzed using multinomial logistic regressions. Phosphorylated tau181 (OR = 1.77, 95% CI: 1.60;1.97), neurofilament light chain (OR = 1.44, 95% CI: 1.31;1.57) and phosphorylated tau181/amyloid-β42 ratio (OR = 1.18, 95% CI: 1.12;1.25) were associated with the highest risk of a dual decline pattern among study participants. In isolated cognitive decliners, robust associations were observed for amyloid-β42, amyloid-β42/40 ratio and glial fibrillary acidic protein, whereas no significant association emerged for those exhibiting only motor decline. Dual decliners present distinct profiles of serum AD biomarkers. The extensive involvement of tau pathology and neurodegeneration suggests an intrinsic biological complexity that necessitates further research, encompassing vascular and neuroinflammatory pathways.

